# Supplementary figures and images for: Survey of SNPs Associated with Total Number Born and Total Number Born Alive in Pig
Source: Genes (Basel). 2020 Apr 30;11(5):491. doi: 10.3390/genes11050491 (PMC7291110; doi:10.3390/genes11050491)

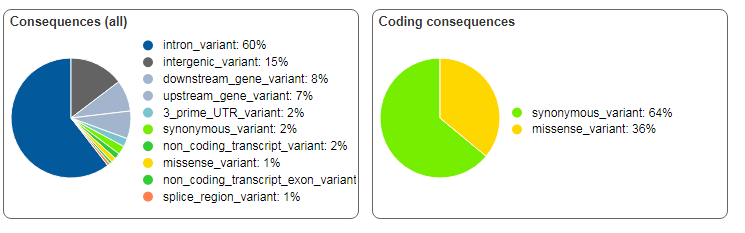

Supplement: Supplementary file 1 [file genes-11-00491-s001.zip › add file 2.docx]
